# On the Quarantine Period for Ebola Virus

**DOI:** 10.1371/currents.outbreaks.2ab4b76ba7263ff0f084766e43abbd89

**Published:** 2014-10-14

**Authors:** Charles N. Haas

**Affiliations:** Departmetn of Civil, Architectural and Environmental Engineering, Drexel University, Philadelphia, Pennsylvania, USA

**Keywords:** ebolavirus, Epidemiology, incubation time, quarantine

## Abstract

Background: 21 days has been regarded as the appropriate quarantine period for holding individuals potentially exposed to Ebola Virus (EV) to reduce risk of contagion. There does not appear to be a systematic discussion of the basis for this period. Methods: The prior estimates for incubation time to EV were examined, along with data on the first 9 months of the current outbreak. These provided estimates of the distribution of incubation times. Results: A 21 day period for quarantine may result in the release of individuals with a 0.2 - 12% risk of release prior to full opportunity for the incubation to proceed. It is suggested that a detailed cost-benefit assessment, including considering full transmission risks, needs to occur in order to determine the appropriate quarantine period for potentially exposed individuals.

## Introduction

For contagious diseases, to reduce the spread, it is necessary and desirable to quarantine each individual who might have been exposed for a sufficient time for either infection to occur or until it can be assured that there is not likely to be infection (and hence spread of contagion). According to the Centers for Disease Control and Prevention^1^:

“When someone has been exposed to a contagious disease and it is not yet known if they have caught it, they may be quarantined or separated from others who have not been exposed to the disease. For example, they may be asked to remain at home to prevent further potential spread of the illness. They also receive special care and observation for any early signs of the illness.”

Parenthetically, if an individual manifests progress into a contagious state, subsequent confinement may occur until the contagiousness ends. The question then arises how long should the quarantine of an individual occur to provide assurance that progress to an infectious state would not result.

Sartwell^2^ conducted one of the first systematic studies on the incubation time for human pathogens and found that a broad spectrum of agents had incubation time distributions that could be modeled as lognormal (although alternative distributional forms were not tested). Leclerc et al. 3 examined the incubation time distribution of a variety of plant pathogens and observed that they could be fit (depending on the pathogen and the plant age) by either the gamma, lognormal, or Weibull distributions. All of these three are skewed right. Williams looked at the theoretical incubation time distribution for pathogens conforming to a stochastic in vivo birth-death process and found that they could also be characterized by a skewed distribution^4^^,^ 5.

In general none of the often used incubation time distributions have a maximum upper limit. In other words

\begin{equation*}\[\bar F\left( t \right) > 0\,\,{\rm{for}}\,{\rm{all}}\,t\]\end{equation*}

Therefore there is no quarantine time that will provide absolute assurance of no residual risk from contagion. Nishiura^6^ pointed out the importance of examining the upper tail of the incubation time distribution when assessing the quarantine period following exposure to smallpox. This was also discussed in the context of the SARS coronavirus outbreak^7^ . Both of these previous authors noted the importance of the distributional form in assessing the upper tail probability, and the influence that data truncation may have on such estimates.

To make use of this approach, an acceptable residual risk needs to be set. To do this, one needs to balance out the costs and benefits of quarantine and risk reduction. For contagious diseases this will require coupling the risk of premature release from quarantine to a disease transmission model such as Legrand et al. 8. This can schematically be shown as in Figure 1. If the costs for enforcing quarantine up to a given time (post exposure) and if the costs associated with releasing individuals at a given time post exposure (with concomitant probability of eventually becoming contagious) can both be estimated, then the optimal quarantine time should be at the intersection of the curves given that both are plotted using the same y-axis. Clearly for pathogens that have a high degree of transmissibility and/or a high degree of severity, the quarantine time should be greater than for agents with lower transmissibility and/or severity. The purpose of this paper is not to estimate where the balancing point should be.

**Schematic of Tradeoff Analysis Needed to Determine Optimum Quarantine Time:**
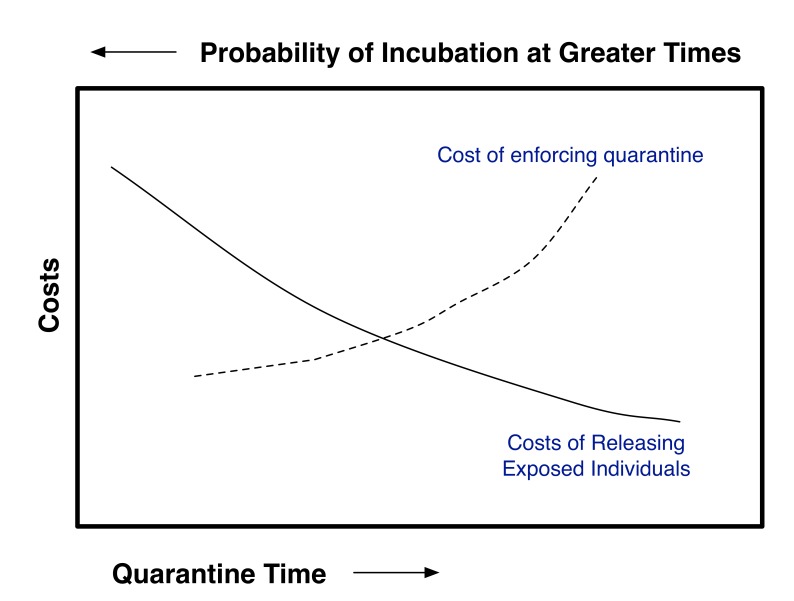

The current operative guidance on quarantine periods for Ebola (Zaire) virus is 21 days, based on WHO assessment that the incubation period is 2–21 days 9. A current review of previous outbreaks cites the same range 10. The precise origin of this assessment is unclear, however it is possibly based on the study of the either the 1976 Zaire outbreak^11^ or 2000 Uganda outbreak^12^ both of which reported (without detailed analysis) a maximum observed incubation time of 21 days.

This contribution will outline what is known about the incubation time distribution for Ebola (Zaire), and what the potential upper tail percentiles might be. With additional information as outlined above, a formal decision analytic approach to deducing the optimal quarantine time may be devised. In view of the current concerns for the Ebola outbreak in West Africa, illustrations using this organism will be presented.

## Prior Knowledge on Ebola Incubation Time Distributions

Breman et al.^11^ did a detailed analysis of the 1976 Zaire outbreak by examining lags between a person-to-person case and the time at which presumed exposure occurred. In their analysis of 109 such cases, they report a mean of 6.3 days and a range of 1 to 21 days, with no distributional fit. However they do report all of the individual observed times.

Chowell et al. 13 analyzed the 1995 Congo and 2000 Uganda outbreaks using an SEIR 14 model. For the S → E → I transition the equations become:

\begin{equation*}\frac{{dS}}{{dt}} =  - \beta(t) SI\\\%0A\frac{{dE}}{{dt}} = \beta(t) SI - kE\\\%0A\frac{{dI}}{{dt}} = kE - \gamma I\end{equation*}

The term kE reflects the conversion rate of exposed to infected. In this model, which has been very frequently used for infectious disease transmission modeling, the implied incubation time distribution is an exponential one with a mean incubation time of 1/k. This can be seen by taking β to zero and observing that the solution to dE/dt is exponential. β is the transmission rate and γ is the rate of exit from the infectious state. The transmission rate may change during the course of an outbreak — e.g., with increasing control mechanisms it may decrease.

Using this analysis Chowell et al. 13 found the mean and standard deviation of the incubation period for the two outbreaks to be:

**Table 1. Incubation Time Mean Estimated by Chowell et al.13:** 

Outbreak	1/k (days) best estimate	standard deviation of the estimate
Congo 1995 (315 Cases)	5.30	0.23
Uganda 2000 (425 Cases)	3.35	0.49

Lekone and Finkenstadt^15^ analyzed the Congo 1995 outbreak (291 cases) using a Bayesian approach and a stochastic SEIR model. Their analysis yielded a larger value for the mean incubation time — 10.11 days using an informative prior. Their approach used an SEIR formulation and thus also an implied exponential distribution.

Eichner et al.^16^ analyzed the 1995 Congo outbreak assuming a lognormal distribution for incubation time (173 cases examined). No assessment of goodness of fit to this distribution was made. They estimated the mean incubation time of 12.7 days with a standard deviation of 4.31 days.

The WHO Response Team^17^ has just published an incubation time distribution based on the first 9 months of the West Africa outbreak (total of 4010 confirmed and probable cases with usable data). They reported a mean incubation period of 11.4 days with an upper 95th percentile of 21 days — and they were able to fit the data to a gamma distribution.

## Results and Conclusions

Figure 2 plots the complementary cumulative distributions from the studies noted above.

**Comparison of Estimated Distributions for EBOV.**
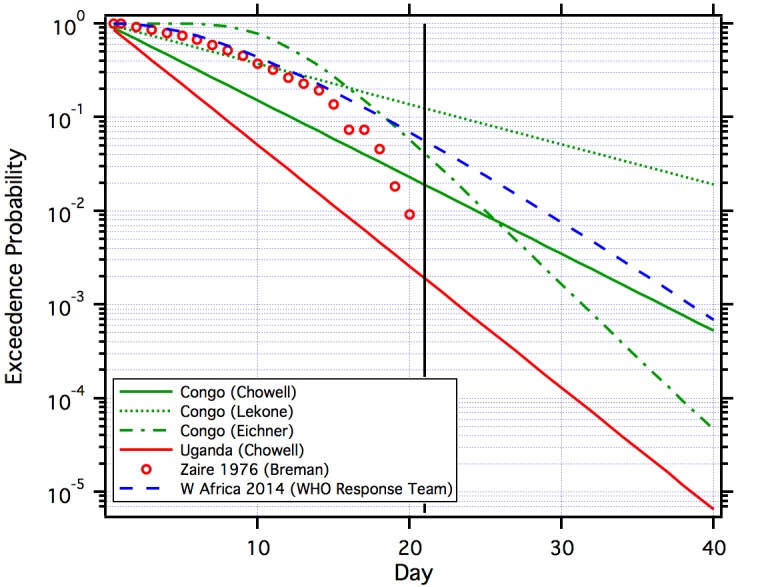
Eichner is lognormal. Breman is the empirical distribution. The W. Africa distribution is gamma. The other distributions are exponential. For reference, a vertical line is shown at 21 days.

Several aspects are clear:

The distribution of incubation times for the Chowell analysis of the Uganda outbreak shows a more rapid incubation time than any of the other outbreaks.
At a 21 day quarantine period, using the data sets other than the Congo analysis of Chowell, three Congo data analyses, the probability of exceedance is between 1.9 and 12%. In other words from 0.1 to 12% of the time, an individual case will have a greater incubation time than 21 days. The 0.1% stems from the 1976 Zaire outbreak, which has the fewest cases analyzed than any of the others in Figure 2.
It is clear that the method of analysis as well as choice of the form of the incubation time distribution are influential.

In discussing the quarantine period for smallpox Nishiura^18^ discussed the use of the upper 95th percentile of the incubation time distribution , although this may vary with the presence of asymptomatic cases and R0 13. If the estimated incubation time distributions for the Congo outbreak (Figure 2) are used, this would suggest a quarantine time of as high as 31 days. But as noted above, the set point (95th, 99th, etc.) for a decision making needs to be based on balancing costs and benefits.

It should also be noted that the functional form used for the incubation time distribution will have an effect on the estimated upper tail probabilities (as well as the mean itself); this was illustrated in the case of SARS 19. This is analogous to the problem of differentiating between distributions in risk assessment^20^. However other candidate skewed distributions should be tested against available data sets.

The focus of the above discussion has been assessing the best estimates of the incubation time distribution in order to develop a rational process for Ebola quarantine estimation. However any estimated distribution has uncertainty, e.g. as shown in Table 1. Therefore the confidence bands of the distribution (e.g., confidence limits to the upper 95th percentile of the incubation time distribution) should be estimated and used in setting a quarantine time.

While the 21 day quarantine value currently used may have arose from reasonable interpretation of early outbreak data, this work suggests a reconsideration is in order and that 21 days may not be sufficiently protective to public health. Further, outbreaks such as the current West Africa EBOV are presenting an opportunity for careful collection of data sufficient to revise and update (perhaps in an adaptive fashion) such recommendations. It may be that incubation time itself is a function of intensity and nature of contact^21^, which may also need to be considered. The estimate of appropriate incubation time would need to explicitly consider the costs and benefits involved in various alternatives, which would incorporate explicit computations from transmission modeling.
